# Laser cooling of antihydrogen atoms

**DOI:** 10.1038/s41586-021-03289-6

**Published:** 2021-03-31

**Authors:** C. J. Baker, W. Bertsche, A. Capra, C. Carruth, C. L. Cesar, M. Charlton, A. Christensen, R. Collister, A. Cridland Mathad, S. Eriksson, A. Evans, N. Evetts, J. Fajans, T. Friesen, M. C. Fujiwara, D. R. Gill, P. Grandemange, P. Granum, J. S. Hangst, W. N. Hardy, M. E. Hayden, D. Hodgkinson, E. Hunter, C. A. Isaac, M. A. Johnson, J. M. Jones, S. A. Jones, S. Jonsell, A. Khramov, P. Knapp, L. Kurchaninov, N. Madsen, D. Maxwell, J. T. K. McKenna, S. Menary, J. M. Michan, T. Momose, P. S. Mullan, J. J. Munich, K. Olchanski, A. Olin, J. Peszka, A. Powell, P. Pusa, C. Ø. Rasmussen, F. Robicheaux, R. L. Sacramento, M. Sameed, E. Sarid, D. M. Silveira, D. M. Starko, C. So, G. Stutter, T. D. Tharp, A. Thibeault, R. I. Thompson, D. P. van der Werf, J. S. Wurtele

**Affiliations:** 1 Department of Physics, College of Science, Swansea University, Swansea, UK; 2 School of Physics and Astronomy, University of Manchester, Manchester, UK; 3 Cockcroft Institute, Sci-Tech Daresbury, Warrington, UK; 4 TRIUMF, Vancouver, British Columbia Canada; 5 Department of Physics, University of California at Berkeley, Berkeley, CA USA; 6 Instituto de Fisica, Universidade Federal do Rio de Janeiro, Rio de Janeiro, Brazil; 7 Department of Physics and Astronomy, University of Calgary, Calgary, Alberta Canada; 8 Department of Physics and Astronomy, University of British Columbia, Vancouver, British Columbia Canada; 9 Department of Physics and Astronomy, Aarhus University, Aarhus, Denmark; 10 Department of Physics, Simon Fraser University, Burnaby, British Columbia Canada; 11 Department of Physics, Stockholm University, Stockholm, Sweden; 12 Department of Physics, British Columbia Institute of Technology, Burnaby, British Columbia Canada; 13 Department of Physics and Astronomy, York University, Toronto, Ontario Canada; 14 Department of Chemistry, University of British Columbia, Vancouver, British Columbia Canada; 15 Department of Physics and Astronomy, University of Victoria, Victoria, British Columbia Canada; 16 Department of Physics, University of Liverpool, Liverpool, UK; 17 Experimental Physics Department, CERN, Geneva, Switzerland; 18 Department of Physics and Astronomy, Purdue University, West Lafayette, IN USA; 19 Soreq NRC, Yavne, Israel; 20 Department of Physics, Ben Gurion University, Beer Sheva, Israel; 21 Physics Department, Marquette University, Milwaukee, WI USA; 22 Faculté de Génie, Université de Sherbrooke, Sherbrooke, Quebec Canada

**Keywords:** Exotic atoms and molecules, Experimental particle physics

## Abstract

The photon—the quantum excitation of the electromagnetic field—is massless but carries momentum. A photon can therefore exert a force on an object upon collision^1^. Slowing the translational motion of atoms and ions by application of such a force^2,3^, known as laser cooling, was first demonstrated 40 years ago^4,5^. It revolutionized atomic physics over the following decades^6–8^, and it is now a workhorse in many fields, including studies on quantum degenerate gases, quantum information, atomic clocks and tests of fundamental physics. However, this technique has not yet been applied to antimatter. Here we demonstrate laser cooling of antihydrogen^9^, the antimatter atom consisting of an antiproton and a positron. By exciting the 1S–2P transition in antihydrogen with pulsed, narrow-linewidth, Lyman-α laser radiation^10,11^, we Doppler-cool a sample of magnetically trapped antihydrogen. Although we apply laser cooling in only one dimension, the trap couples the longitudinal and transverse motions of the anti-atoms, leading to cooling in all three dimensions. We observe a reduction in the median transverse energy by more than an order of magnitude—with a substantial fraction of the anti-atoms attaining submicroelectronvolt transverse kinetic energies. We also report the observation of the laser-driven 1S–2S transition in samples of laser-cooled antihydrogen atoms. The observed spectral line is approximately four times narrower than that obtained without laser cooling. The demonstration of laser cooling and its immediate application has far-reaching implications for antimatter studies. A more localized, denser and colder sample of antihydrogen will drastically improve spectroscopic^11–13^ and gravitational^14^ studies of antihydrogen in ongoing experiments. Furthermore, the demonstrated ability to manipulate the motion of antimatter atoms by laser light will potentially provide ground-breaking opportunities for future experiments, such as anti-atomic fountains, anti-atom interferometry and the creation of antimatter molecules.

## Abstract (web summary)

The successful laser cooling of trapped antihydrogen, the antimatter atom formed by an antiproton and a positron (anti-electron), is reported.

## Main

The antihydrogen atom, the simplest example of atomic antimatter, offers unique opportunities in challenging the foundational framework of contemporary physics. Precision comparisons of antihydrogen’s properties with those of the well studied hydrogen atom allow tests of fundamental symmetries such as charge–parity–time invariance and Einstein’s equivalence principle, which underpin quantum field theory and the general theory of relativity. The field of antihydrogen studies has seen tremendous advances in recent years. Techniques have been developed to produce^15–18^, confine^19–21^ and interrogate cold antimatter atoms with microwaves^13,22^ and lasers^10–12,23^. In addition, experiments are being built to measure the gravitational properties of antimatter^14,24,25^. The finite kinetic energies of the anti-atoms impose substantial limitations on the precision of many of these measurements. Therefore, preparation of antihydrogen at the lowest possible kinetic energies is an important objective in the field.

Doppler cooling, the type of laser cooling used in this work, takes place via the velocity-dependent absorption of near-resonant photons by atoms. The atoms moving towards the photon source are selectively excited by setting the photon frequency slightly below the resonance for the atom at rest (red detuning), resulting in a net force opposing the motion^2,3^. The spontaneous emission of a photon from the excited atom occurs in a spatially symmetric manner in free space, resulting in a null average recoil force. In the case of (anti)hydrogen^26^, by exciting the 1S–2P Lyman-α transition, a net velocity change of 3.3 m s^−1^ can be exerted on average by each 121.6-nm photon scattered. In principle, repeating such scatterings only a few dozen times should slow (anti)hydrogen atoms, initially trapped in a well depth of about 50 μeV (corresponding to a maximum speed of about 90 m s^−1^), down to submicroelectronvolt energies.

In practice, however, laser cooling of antihydrogen presents a number of technical challenges. First, generating and transporting radiation at 121.6 nm is difficult. There are no convenient lasers or nonlinear crystals at vacuum ultraviolet wavelengths, and the light is readily attenuated in air and in optical components. Second, the experimental requirements of antihydrogen experiments severely restrict optical access to the anti-atoms. Because they need to be synthesized from their antiparticle constituents and trapped in situ, extensive infrastructure is required, limiting the available space. Third, the currently available maximum density (about 1 cm^–3^) of the scarce anti-atoms—more than 10 orders of magnitude lower than the 10^11^–10^14^ cm^–3^ in previous trapped hydrogen experiments^26,27^—results in extremely low rates of laser transitions. Furthermore, such a low density rules out collisions as an equilibration mechanism for achieving three-dimensional cooling with one-dimensional laser access, as was done for the pioneering work on laser cooling of hydrogen^26^ (Methods).

Despite these challenges, the feasibility of laser cooling antihydrogen in the Antihydrogen Laser Physics Apparatus (ALPHA) using a pulsed laser was explored in ref. ^9^. Simulations showed that the low excitation rate could be overcome with long cooling duration, enabled by the previously observed long confinement time^20^. (The excitation rate can also be increased by a higher repetition rate of the laser.) The simulations also predicted that three-dimensional cooling could be achieved with one-dimensional laser access by anharmonic coupling of antihydrogen’s motional degrees of freedom in the trap, an effect that could be enhanced by tailoring the profile of the magnetic trapping field.

To realize this goal, we have developed a novel, solid-state-based, pulsed Lyman-α laser^28^, and previously used it to probe the 1S–2P transition in trapped antihydrogen^10^ and to observe the fine structure and the Lamb shift^11^. These studies established our capability to precisely drive and detect the Lyman-α transition with single-atom sensitivity. Meanwhile, we have dramatically improved antihydrogen production and trapping techniques^29^ and developed techniques to drive the 1S–2S transition^12^. The demonstration of laser cooling of antihydrogen atoms and its application to 1S–2S spectroscopy reported here represent the culmination of these developments over recent decades.

## Experimental setup

**Fig. 1:**
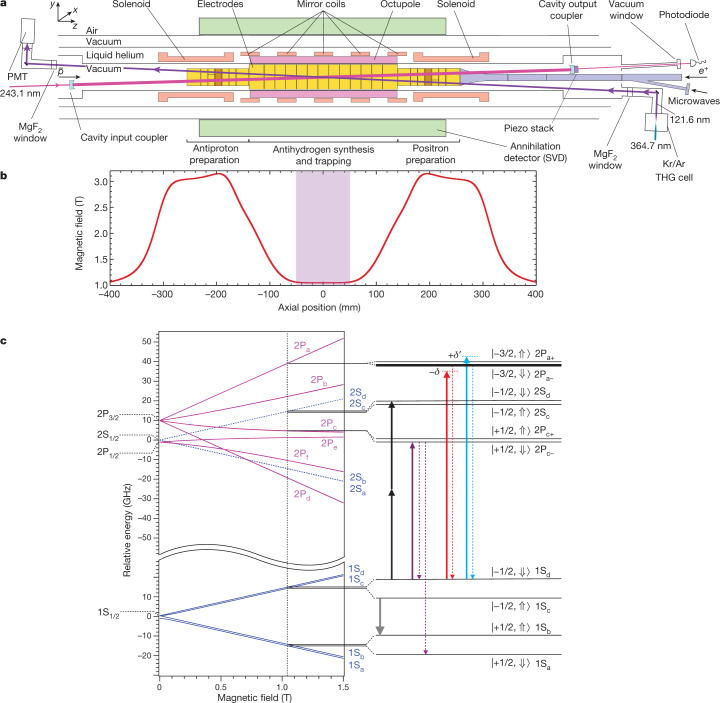
The ALPHA-2 apparatus schematic and antihydrogen energy levels. **a**, Central parts of the ALPHA-2 apparatus are schematically shown. The field for the magnetic minimum trap is produced by five mirror coils for longitudinal confinement and one octupole coil for transverse confinement. The trap has a depth of about 50 μeV with an axial length of 280 mm and a diameter of 44.35 mm. The magnetic trap is superimposed on a cryogenic Penning trap (the electrodes are shown in yellow). An external solenoid, not shown, provides a 1-T base field for charged particle trapping and cooling. The solenoids at either end of the trap further boost the field in the preparation traps to 3 T for more efficient cyclotron cooling of electrons, positrons (*e*^+^) and antiprotons ($\bar{p}$), before antihydrogen synthesis. The atom trap is surrounded by a silicon vertex annihilation detector made of three layers of double-sided microstrip sensors. The pulsed Lyman-α light at 121.6 nm, generated in a gas cell immediately outside the ultrahigh vacuum chamber, is introduced through a magnesium fluoride window with an angle of 2.3° with respect to the trap axis to allow particle loading on axis into the Penning trap. The intensity of the 121.6-nm pulse is recorded by a solar-blind photomultiplier (PMT) placed after the trap. A cryogenic optical cavity serves to both build up the 243.1-nm laser light needed to drive the 1S–2S transitions, and to provide the counter-propagating photons that cancel the first-order Doppler shift. Microwaves, used to drive hyperfine transitions, and to perform electron cyclotron resonance magnetometry, are injected through the microwave guide. According to the coordinate system shown, we define the longitudinal kinetic energy to be 1/2*m*_H_${v}_{z}^{2}$, and the transverse one to be 1/2*m*_H_ $({{v}_{x}}^{2}+{{v}_{y}}^{2})$, where *m*_H_ is the mass of antihydrogen, and *v*_*x*_, *v*_*y*_ and *v*_*z*_ are the velocity components in the *x*, *y* and *z* directions. **b**, Magnetic field profile on the axis of the trap. The shaded region illustrates a volume in which the field on axis is uniform to 0.01 T, corresponding to a Zeeman shift of 140 MHz in the 1S–2P_a_ transition. Immediately before reach run, the magnetic field at the centre of the trap was measured via electron cyclotron resonance and the laser frequencies were adjusted accordingly. The measured magnetic minimum field, averaged over the pre-run measurements, was 1.03270 ± 0.00007 T, where the error is the standard deviation from the set of measurements. **c**, The energy levels of the antihydrogen in the *n* = 1 and *n* = 2 states are depicted as a function of the magnetic field. On the vertical axis, the centroid energy difference, *E*_1S–2S_ = 2.4661 × 10^15^ Hz, has been suppressed. The dotted vertical black line represents the field at the magnetic minimum of our trap, 1.0327 T (see above). Details of the energy levels near this field and their state labels are shown on the right of the figure. The first value in the ket notation represents the quantum number of the projection of the total angular momentum of the positron, *m*_*L*_ + *m*_*S*_, where *L* is the orbital angular momentum (*L* = 0 for the S state and *L* = 1 for the P state, respectively) and *S* is the spin (*S* = 1/2). The double arrow shows the antiproton spin (up or down). Initially, both the 1S_c_ and 1S_d_ states are trapped in our magnetic trap. The grey arrow indicates the microwave-driven 1S_c_ → 1S_b_ transition to eliminate the anti-atoms in the 1S_c_ hyperfine state and prepare a doubly spin-polarized antihydrogen sample in the 1S_d_ state. The solid and broken red (cyan) arrows indicate the cycling transition for laser cooling (heating) with red (blue) detuning −*δ* (+*δ*′). The purple arrow represents the probe laser excitation to the 2P_c–_ level. Note that the 2P_c_ state at a magnetic field of about 1 T is a superposition of the positron spin-up (*m*_*L*_ = 0, *m*_*S*_ = +1/2) and spin-down (*m*_*L*_ = +1, *m*_*S*_ = –1/2) states. Owing to this superposition, upon de-excitation from the 2P_c_ state, the anti-atom can either go back to the original 1S_d_ state, or undergo an effective ‘spin flip’ transition to the 1S_a_ state. In the latter case, the anti-atom is forced out of the trap and detected via its annihilation signal. The black arrows show the two-photon excitation from the 1S_d_ state to the 2S_d_ state.

The ALPHA-2 apparatus (Fig. 1a, b) is a second-generation antihydrogen trapping experiment located at CERN. The production and trapping of antihydrogen for this work generally follows the procedure detailed in ref. ^29^. Briefly, antihydrogen atoms are created in a Penning trap by mixing about 10^5^ antiprotons from CERN’s antiproton decelerator^30^ with 3 × 10^6^ positrons from an accumulator^31^. Typically, 10–30 anti-atoms are magnetically confined^19,20^ in an overlapping magnetic minimum trap in each 4-min production cycle. Such cycles are repeated over several hours to accumulate about 1,000 anti-atoms in a procedure referred to as ‘stacking’^29^. The magnetic trap is surrounded by a silicon vertex detector (SVD)^32,33^, which monitors antihydrogen annihilations with a high efficiency and low background, with the help of a machine-learning-based analysis procedure (Extended Data Table 1, Methods)^34,35^. Important features of ALPHA-2 (compared with the original ALPHA device) include the incorporation of optical access to the trapped antihydrogen (at both ultraviolet and vacuum ultraviolet wavelengths) and doubling of the number of superconducting coils (from four to eight). The latter permits flexible configuration of the trapping field profile, allowing increased laser excitation rates by reducing the field inhomogeneity near the trap centre. Importantly, it also helps promote the mixing of the antihydrogen motional degrees of freedom by enhancing the anharmonicity of the trapping potential^9,36^.

The pulsed 121.6-nm Lyman-α radiation was produced in a krypton/argon gas medium via third-harmonic generation (THG) of 364.7-nm light, which in turn was obtained in an all-solid-state laser system via frequency doubling of pulse-amplified, continuous-wave-seeded 729.4-nm pulses^10,11,28^ (Methods). The pulsed THG scheme was chosen^28^ over other alternatives^37,38^ for its technical advantages, including the robustness in operation. A similar laser system has been reported by another group^39^. The 121.6-nm pulses were linearly polarized, and had a duration of 15 ns (full-width at half-maximum of laser intensity) at a 10-Hz repetition rate; hence the duty factor was 1.5 × 10^−7^. The laser bandwidth at 121.6 nm was estimated to be between 50 MHz and 100 MHz, on the basis of measurements and numerical modelling.

The energy of the 121.6-nm laser pulse inside the trap ranged from 0.3 nJ to 3.5 nJ, as measured by a calibrated photomultiplier after passage through the trap. The laser beam radius (1/e of the electric field distribution) was about 3.6 mm in the interaction region. The frequency of the 729.4-nm seed laser was locked to a high-precision wavemeter.

Figure 1c shows the expected energy levels of antihydrogen in a magnetic field. Doppler cooling is performed by repeatedly driving single-photon transitions between the 1S state and the 2P_a_ state (one of the Zeeman sublevels of the 2P_3/2_ state) with a laser frequency that is slightly red-detuned from the resonance of the atom at rest. In a strong magnetic field, this is a closed cycling transition, hence suitable for cooling. Conversely, Doppler ‘heating’ can be achieved with light at a slightly blue-detuned frequency. As our magnetic minimum trap confines both the 1S_c_ and 1S_d_ states, to simplify the diagnostics, we prepare a doubly spin-polarized, that is, hyperfine-purified, sample of 1S_d_ atoms, by removing most of the 1S_c_ atoms via resonant microwaves^11,22^ (Methods). (By doubly spin-polarized, we refer to the fact that both positron and antiproton spins are polarized in a sample.) Hence, essentially only the 1S_d_ → 2P_a–_ transition is excited during laser cooling.

Single-photon excitations from 1S_d_ to the 2P_c–_ and the 2P_f–_ fine-structure states were observed in our previous studies^10,11^. These excited states, which are superpositions of positron spin-up and spin-down states, can de-excite to the ground state with either positron spin state, 1S_a_ or 1S_d_ (Fig. 1c). The transition to the untrappable 1S_a_ state (a process we call ‘spin flipping’) effectively reverses the positron spin direction, leading to the loss of anti-atoms from the magnetic trap. These transitions are therefore not desirable for laser cooling. However, they can be used to probe the sample’s energy distribution via the detection of the annihilation signals of the spin-flipped anti-atoms, discussed below. Of the two possible fine-structure levels (2P_c–_ and 2P_f–_) for such diagnostic measurements, we focus on driving the 1S_d_ → 2P_c–_ transition, as it has a smaller Zeeman slope (−16 MHz mT^−1^)—hence a smaller Zeeman broadening—than that of the 1S_d_ → 2P_f –_ transition (−26 MHz mT^−1^) at 1 T (Fig. 1c). It is also closer in frequency to the cooling transition, allowing a simpler laser control protocol.

Finally, using counter-propagating, continuous-wave, 243.1-nm laser light in a resonant enhancement cavity surrounding the trapping volume, we can drive the Doppler-free 1S_d_–2S_d_ transition^12,23^ (black arrows in Fig. 1c). The laser-induced transition is identified by the detection of the annihilation on trap walls, coming from photoionized antiprotons or spin-flipped antihydrogen^12,40^. The 243.1-nm laser system and its frequency stabilization method are described in refs. ^12,23^.

## Experimental protocol

A total of four measurement series were performed to study the laser-cooling process, as summarized in Table 1. We refer to these measurements as the ‘cooling’ experiment (as opposed to the ‘spectroscopy’ experiment below). Each series consisted of one to three runs, which typically proceeded in the following manner. (1) A stacking phase: production and accumulation of roughly 500 to 1,000 antihydrogen atoms over a period of 2 h to 4 h. (2) A hyperfine polarization phase: resonant microwaves were applied for 32 s to force the 1S_c_ state atoms out of the trap, resulting in a doubly spin-polarized sample of antihydrogen (Methods). (3) A cooling/heating phase: the 1S_d_ → 2P_a–_ transition was driven for 2 h to 4 h by exposure to the 121.6-nm laser with its frequency detuned approximately to either +150 MHz, +170 MHz (series 2, heating) or –240 MHz (series 3, cooling). Detuning frequencies were measured relative to the atom’s at-rest resonant frequency at the magnetic minimum of our trap, the field of which was measured in situ via the electron cyclotron resonance method^41,42^ before the start of every run (Fig. 1b). Data were also collected with no cooling laser applied (series 1, no laser). (4) A probing phase: the 1S_d_ → 2P_c–_ transition was driven at nine frequencies ranging from –1.1 GHz to 0.9 GHz relative to the resonant frequency for a duration of 2 h to 4 h to characterize the cooled/heated antihydrogen atoms via the detection of the spin-flip transitions^10^. The laser frequency was changed every 50 s in a non-monotonic fashion to mitigate effects related to the depletion of the sample of antihydrogen. (5) A release phase: any remaining atoms were counted by shutting down the magnetic trap, typically in about 2 s. We also performed a measurement referred to as ‘stack and cool’ (series 4). In this series, laser cooling was applied continuously throughout the 5-h stacking phase—during which about 1,100 antihydrogen atoms were accumulated through 75 production cycles—in addition to the standard cooling phase, which followed stacking and lasted 6 h. Note that the duration of each phase of our experimental protocol was dictated by the practical limitations of receiving the antiproton beam for only several hours per day, and requiring time for the preparation of the measurement on the following day.

**Table 1:** Experimental dataset

Series	Type	1S_d_ → 2P_a–_ detuning (MHz)	Stacking phase		Cooling/heating phase		Probing phase	
			Number of stacks (approximate time)	Average pulse energy (nJ)	Number of pulses (approximate time)	Average pulse energy (nJ)	Number of pulses (approximate time)	Average pulse energy (nJ)
1	No laser	NA	30 (2 h)	NA	NA (No wait)	NA	72,000 (2 h)	1.50
2	Heating	+150	28 (2 h)	NA	72,000 (2 h)	3.5	72,000 (2 h)	0.84
3	Cooling	–240	60 (4 h)	NA	144,000 (4 h)	2.2	144,000 (4 h)	0.46
3	Cooling	–240	60 (4 h)	NA	144,000 (4 h)	1.9	144,000 (4 h)	0.65
2	Heating	+150	30 (2 h)	NA	144,000 (4 h)	1.7	144,000 (4 h)	0.47
2	Heating	+170	60 (4 h)	NA	144,000 (4 h)	1.2	144,000 (4 h)	0.34
1	No laser	NA	59 (4 h)	NA	NA (4 h wait)	NA	129,600 (3.6 h)	0.39
4	Stack and cool	–230	75 (5 h)	1.9	216,000 (6 h)	1.6	126,000 (3.5 h)	0.37
B	1S–2S No cooling	NA	150 (11.5 h)	NA	NA (no wait)	NA	NA (1.5 h)	1.3 W at 243.1 nm
A	1S–2S Stack and cool	–220	130 (9 h)	1.8	216,000 (6 h)	2.1	NA (1.8 h)	1.3 W at 243.1 nm

A list of experimental parameters for each run in the experimental series are tabulated in chronological order for the cooling experiment (series 1–4) and the spectroscopy experiment (series A and B). For series 1–4, the average pulse energy represents an estimated pulse energy of the 121.6-nm laser inside the trap. For the probing phase of series A and B, we list an estimated continuous-wave, build-up power of the 243.1-nm laser in the cavity surrounding the trap. NA, not applicable.

The number of annihilation counts of the spin-flipped antihydrogen atoms, detected in each phase of the cooling experiment, is listed in Extended Data Table 2 and discussed further in Methods. The results reported here concern the 85% to 95% of the 1S_d_ anti-atoms that underwent spin-flip probe transitions, and, unless otherwise stated, we do not consider the remaining unprobed populations.

In addition, we collected two series of data, where laser-cooled (run A) and uncooled (run B) samples were subjected to counter-propagating 243.1-nm laser light to drive the two-photon 1S–2S transition. These runs are referred to as the ‘spectroscopy’ experiment and are discussed below.

## Results

**Fig. 2:**
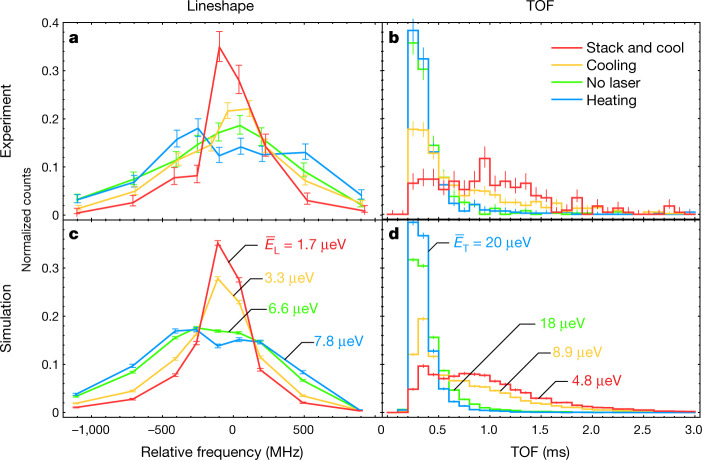
Laser cooling of antihydrogen. The spectral lineshapes and the TOF distributions, obtained during the probing phase by detecting antihydrogen annihilations resulting from laser-induced spin flips. In all cases, the curves are drawn to guide the eye. **a**, The experimental lineshapes given by the number of annihilation counts within a TOF time window of 0 to 3 ms, as a function of the probe laser frequency relative to the resonant frequency. **b**, TOF distributions representing the time between the nanosecond-scale probe laser pulse and the detection of the annihilation. Events with an axial annihilation position between +10 cm and –10 cm are plotted. The distributions are compared for the experimental series given in Table 1: the no-laser series (green); the heating series with a detuning of approximately +160 MHz (blue); the cooling series with a –240-MHz detuning (orange); and the ‘stack and cool’ series where a –230-MHz detuning was applied during both the stacking phase and the cooling phase (red). **c**, **d**, The corresponding simulations for the lineshapes (**c**) and the TOF distributions (**d**). Each distribution is normalized to its total number of counts, and the error bars represent 1 s.d. counting statistical uncertainties. The values labelled ${\bar{E}}_{{\rm{L}}}$ and ${\bar{E}}_{{\rm{T}}}$ represent the mean of ‘true’ longitudinal and transverse energies, respectively, of the simulated atoms at the time of the spin-flip transitions. See text and Methods.

We first focus on the cooling experiment (series 1–4), and present experimental evidence for laser cooling in Fig. 2a, b. Figure 2a shows the observed spectral lineshapes of the 1S_d_ → 2P_c–_ transition, during the probing phase. The plots depict the distributions of detected annihilations from laser-induced spin flips, measured as a function of the probe laser frequency relative to the expected resonance. Each distribution is normalized to its total number of counts. The estimated uncertainty in our laser frequencies during the experiment is 54 MHz at 121.6 nm (Methods). Figure 2a shows that the application of a blue-detuned laser broadens the spectral lineshape (curve labelled heating), while the red-detuned laser (cooling) narrows it. Particularly noticeable is the narrow peak—with a root mean square (r.m.s.) width of 0.40 ± 0.03 GHz—for the ‘stack and cool’ series, which had the longest effective cooling time. In comparison, the no-laser series has a width of 1.1 ± 0.1 GHz. The observed narrowing of the spectral lines in the red-detuned series reflects the reduction in the component of the antihydrogen velocity parallel to the laser beam—essentially along the trap longitudinal axis—due to Doppler broadening having been reduced via laser cooling (Methods).

Figure 2b depicts the distributions of the time of flight (TOF)—the time delay between the application of the nanoseconds-long 1S_d_ → 2P_c–_ probe laser pulse and the detection of the associated annihilation. The time resolution of the TOF measurement is of the order of 1 μs. The TOF provides information on the transverse speed ${({{v}_{x}}^{2}+{{v}_{y}}^{2})}^{1/2}$, perpendicular to the trap longitudinal axis *z*, of an anti-atom that travelled from the laser excitation point to the trap walls^10^ (Methods). Here, *v*_*x*_ and *v*_*y*_ are the atom’s velocities perpendicular to *z* (Fig. 1a). While the effect of the blue-detuned laser on the TOF distribution (heating) is not evident, the application of the red-detuned laser (cooling) clearly shifts the distribution to later times. Again, the ‘stack and cool’ series shows a dramatic shift; compared with the no-laser series, the mean of the TOF is shifted from 0.42 ± 0.01 ms to 1.00 ± 0.03 ms—an indication that a substantial reduction in the transverse velocity of the anti-atoms has been achieved by the application of cooling pulses. See Methods for a further discussion on laser heating.

Importantly, with the red-detuned laser applied, both the longitudinal and transverse velocities are reduced; this suggests that three-dimensional cooling is realized despite having essentially only one-dimensional laser access. Cooling in the transverse plane is presumably enabled via the coupling of antihydrogen’s motional degrees of freedom in the anharmonic magnetic trapping potential^9,36,43^. See Methods for a discussion on the dimensionality of cooling in our experiment.

## Comparison with simulations

We have compared the results of our cooling experiment to numerical simulations to gain insights into the cooling process. The simulation tracks the motion of trapped antihydrogen atoms and their interactions with the cooling and probing laser radiation (Methods). In this work, we have empirically adjusted one input parameter, *W*_cool_—the total amount of energy injected inside the trap by the cooling (or heating) laser—to match the experiment. The probing laser energy is unchanged in simulation. With this single parameter *W*_cool_ adjusted, the simulations reproduce the qualitative features of our experimental results. The need for this scaling probably reflects our imperfect understanding of some parameters of the experiment (as detailed in Methods), and is a subject of future work. However, this does not affect our primary conclusions.

Figure 2c, d shows the lineshapes and TOF distributions obtained in the simulations, which are in a qualitative agreement with the experiment. The actual or ‘true’ energies—as opposed to the reconstructed energies, as discussed below—of the simulated antihydrogen atoms at the time of probing spin-flip transitions are denoted as *E*_L_ and *E*_T_, for the longitudinal and transverse energies, respectively. In Fig. 2c, d, we give the values ${\bar{E}}_{{\rm{L}}}$ and ${\bar{E}}_{{\rm{T}}}$, representing the mean values of *E*_L_ and *E*_T_. We observe that between the no-laser and the ‘stack and cool’ simulations, both ${\bar{E}}_{{\rm{L}}}$ and ${\bar{E}}_{{\rm{T}}}$ are reduced by roughly a factor of 4. While the antihydrogen distributions are highly non-thermal as discussed below, we note that a thermal distribution with the same average kinetic energy as the simulated ‘stack and cool’ sample would have a temperature of about 50 mK. The approximate agreement of the experimental distributions (Fig. 2a, b) with the simulations (Fig. 2c, d) implies that similar reductions in antihydrogen mean energies have been attained experimentally.

## Reconstruction of antihydrogen energies

**Fig. 3:**
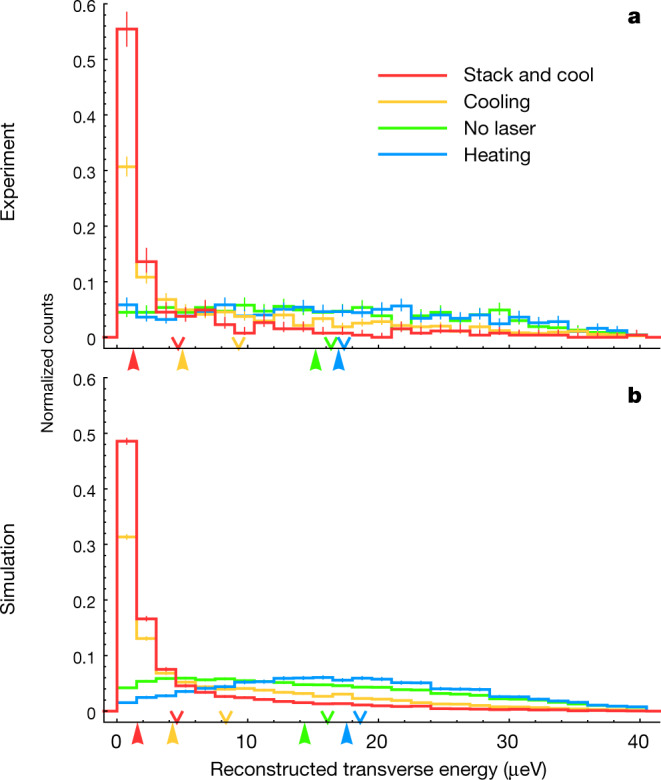
Reconstructed transverse energies of the laser-cooled and heated antihydrogen. **a**, Distributions of the transverse kinetic energies reconstructed from the TOF of antihydrogen for different series. On the horizontal axis, the mean values of the reconstructed energies for each series are marked by downward-facing arrows, and the medians by upward-facing arrows. **b**, Corresponding simulations, where simulated events are analysed in the same way as above. The error bars represent 1 s.d. statistical uncertainties. See text and Methods.

**Extended Data Fig. 1:**
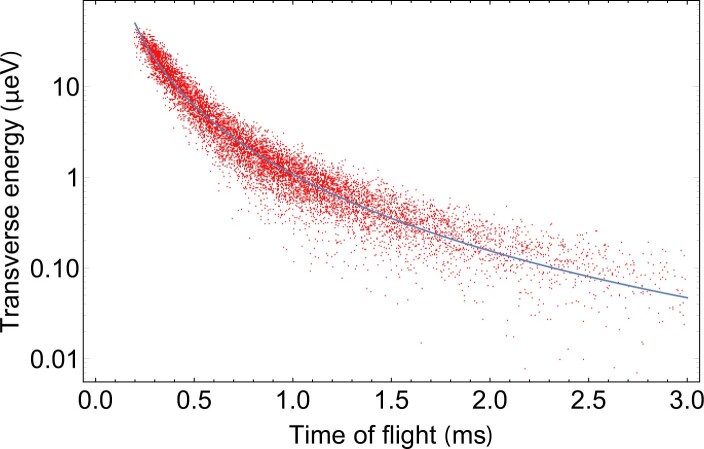
Comparison of the reconstructed and true transverse energies in simulated events. Each red point shows a simulated antihydrogen’s true transverse energy at the time of the spin flip, plotted against its TOF. The simulation was performed for the conditions of the ‘stack and cool’ series. The true energies can be compared with the reconstructed transverse energies (blue curve), derived from the TOF using the one-dimensional model. The mean of the true energies agrees to within 10% with that of the reconstructed energies. See Methods.

To further quantify the extent of cooling, we focus on the transverse kinetic energy of the anti-atoms, for which accurate experimental determination is possible on the basis of the TOF diagnostic. Plotted in Fig. 3a (Fig. 3b) are distributions of transverse kinetic energies, reconstructed from the recorded (simulated) TOF by solving the equation of motion of the spin-flipped anti-atoms in the trap magnetic field (Methods). Here simplifying assumptions—that the spin flips take place on the trap axis, and that the trapping field strength is a function only of the radial distance from the axis—enable event-by-event conversion of the TOF to the transverse energy. These are good approximations to our conditions (Extended Data Fig. 1, Methods). Notably in Fig. 3a, a striking low-energy peak is developed in the laser-cooled samples. Compared with the no-laser series, the growth in the population below 1 μeV in the ‘stack and cool’ series is more than a factor of 10, from 3.2 ± 0.8% to 43 ± 3%. The mean of the reconstructed energies in the experiment (down arrows in Fig. 3a) is reduced from 16.3 ± 0.5 μeV to 4.7 ± 0.5 μeV between the no-laser and ‘stack and cool’ series. This reduction is consistent with that for ${\bar{E}}_{{\rm{T}}}$, the mean of true energies of the simulated events, given in Fig. 2d. However, the median value of the reconstructed energies (up arrows) is decreased even further—from 15.1 ± 0.8 μeV to 1.3 ± 0.1 μeV, that is, by more than an order of magnitude. These characteristics in the transverse energy distributions reflect the highly non-thermal nature of our dilute antihydrogen samples, where collisions are negligible. Similar features are obtained in Fig. 3b, where the simulation events are analysed in the same way as the experiment.

**Extended Data Fig. 2:**
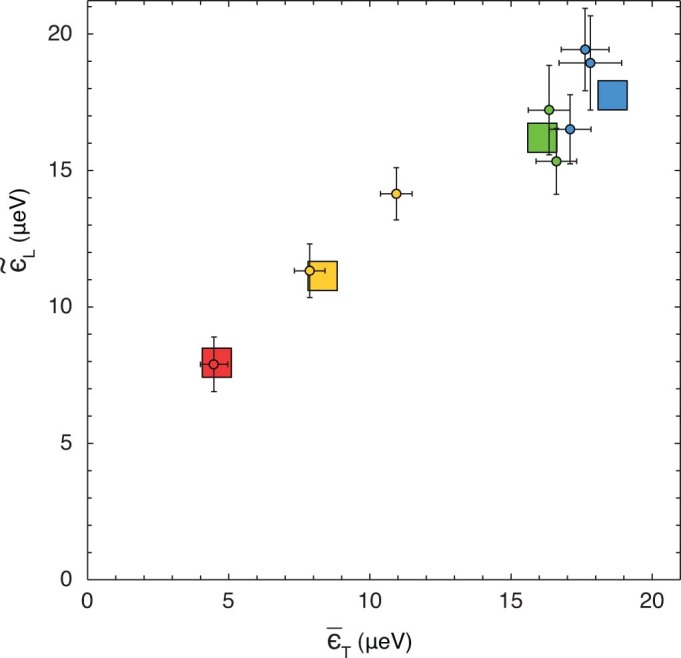
Sample-by-sample correlations between the longitudinal and transverse motions of antihydrogen in the cooling experiment. The correlations between the longitudinal and the transverse energies, as represented by the parameters ${\tilde{\epsilon }}_{{\rm{L}}}$ and ${\bar{\epsilon }}_{{\rm{T}}}$, are shown for the eight experimental runs (circles with error bars). The corresponding simulations for four series (squares) are also shown, for which the simulated data are analysed in the same way as the experimental data. ${\tilde{\epsilon }}_{{\rm{L}}}$ is an estimated upper limit on the average longitudinal kinetic energy of the sample and ${\bar{\epsilon }}_{{\rm{T}}}$ is the mean of the reconstructed transverse kinetic energies given in Fig. 3a, b. See Fig. 2 for the colour code for the series. The error bars represent 1 s.d. statistical uncertainties. These correlations corroborate the observation that antihydrogen atoms are cooled in both longitudinal and transverse degrees of freedom.

In principle, we could also reconstruct the longitudinal energies from the spectral lineshapes. However, the existence of a multitude of line-broadening mechanisms, as well as the limited number of frequency points, would complicate such analysis and prevent straightforward reconstruction of the longitudinal energies. In this work, we instead derive an upper limit of the mean longitudinal energy ${\tilde{\epsilon }}_{{\rm{L}}}$ as an approximate measure of cooling (Methods). The evolution of ${\tilde{\epsilon }}_{{\rm{L}}}$ over the experimental series agrees qualitatively with the prediction of simulations, corroborating the observation of three-dimensional cooling (Extended Data Fig. 2).

## Correlation between longitudinal and transverse energies

**Fig. 4:**
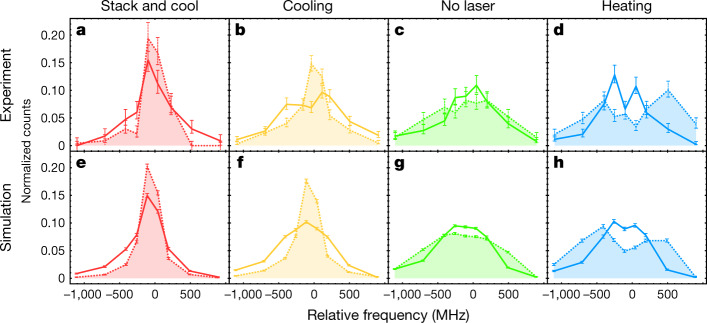
Comparison of spectral lineshapes between transversely cold and hot anti-atoms within the same series in the cooling experiment. **a**, Comparison of the spectral lineshapes between equally sized subsamples of the ‘stack and cool’ series data. The lineshape for the subsample with the transverse energy greater (smaller) than its median value is shown with a solid line (dashed line filled under the curve). **b**–**h**, Analogous comparisons are given for the cooling (**b**), no-laser (**c**) and heating (**d**) series, and the corresponding simulations (**e**–**h**). The error bars represent 1 s.d. counting statistics. In all cases, the curves are drawn to guide the eye. These correlations indicate that in the laser-cooling series (**a**, **b**), transversely colder atoms are also longitudinally colder, while the correlation is reversed for the heating series (**d**). See text and Methods.

**Extended Data Fig. 3:**
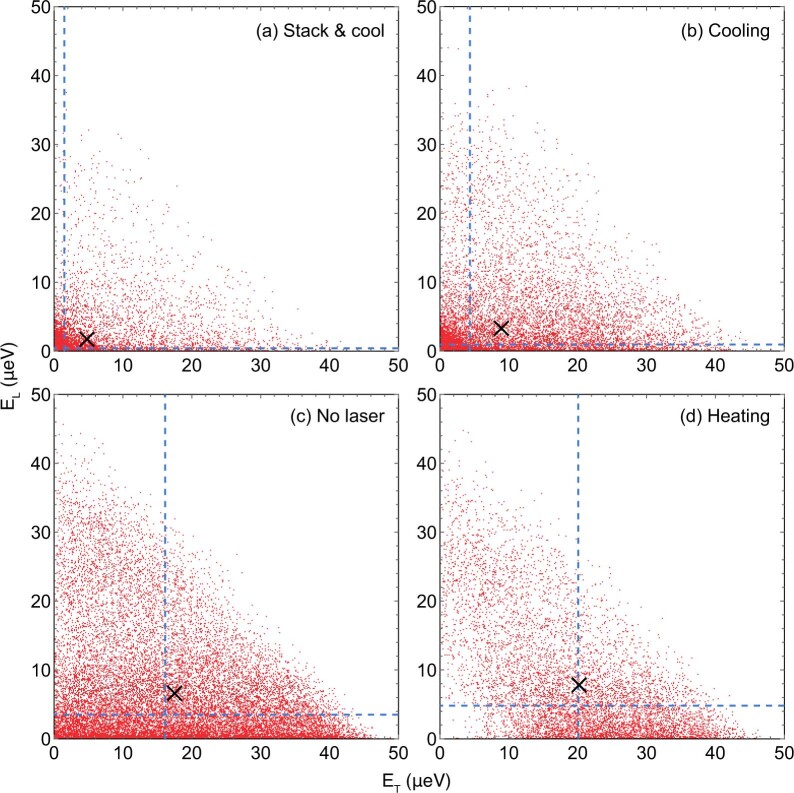
Simulated correlations between the longitudinal and transverse energies within the same samples in the cooling experiment. **a**–**d**, Red dots show the values of true longitudinal (*E*_L_) and transverse (*E*_T_) energies of simulated antihydrogen atoms at the time of spin flip, for the four different series. The blue dashed lines depict the median longitudinal and transverse energies (cf. Fig. 4) and the black crosses indicate their mean values. Note the appearance of an intense peak near *E*_L_ and *E*_T_ ≈ 0, in the ‘stack and cool’ (**a**) and cooling (**b**) series, compared with the no-laser series (**c**), indicating strong three-dimensional cooling. The distributions show that the total energy, *E*_L_ + *E*_T_ is bound by the trap depth of about 50 μeV. This results in anti-correlation between *E*_L_ and *E*_T_ for the atoms with a total energy comparable to the trap depth. This anti-correlation is enhanced in the heating series (**d**), where the population of the coldest atoms (near *E*_L_ and *E*_T_ ≈ 0) are removed by the laser heating. These features in the simulated events qualitatively explain the observations in Fig. 4.

In Fig. 4, we examine the correlation of the longitudinal and transverse energies within the same samples in the cooling experiment. The lineshape distributions are compared by dividing the data from each series into two equally sized subsamples on the basis of whether the reconstructed transverse energies are greater or less than their median. The ability of the TOF diagnostic to provide event-by-event (that is, atom-by-atom) information permits such selection on the basis of the transverse energy of the individual anti-atom. Qualitative features in the experimental curves (Fig. 4a–d) are reproduced by the simulations (Fig. 4e–h). For both the ‘stack and cool’ and the cooling series, the lineshapes are narrower for the subsample with smaller reconstructed transverse energies (dashed filled curves in Fig. 4a, b, with r.m.s. widths of 217 ± 28 MHz and 284 ± 15 MHz, respectively), compared with those for larger transverse energies (solid curves, with widths 284 ± 15 MHz and 371 ± 17 MHz). This indicates that the longitudinally cooled population is also cooled transversely, implying that individual atoms are cooled in three dimensions. (In contrast, Extended Data Fig. 2 shows that the ensemble average is cooled.) Interestingly, in the heating series (Fig. 4d), the energy correlation is reversed from the laser-cooling series, that is, the transversely colder anti-atoms appear to be longitudinally hotter with an r.m.s. lineshape width of 503 ± 21 MHz (dashed filled curve), compared with the transversely hotter subsample (359 ± 20 MHz, solid curve). This trend can be understood by examining the simulated correlations (Extended Data Fig. 3). Recall that our magnetic trap can stably confine only the anti-atoms with the sum of the longitudinal and transverse energies less than the trap depth of about 50 μeV. Hence, for the atoms with total energy comparable to the trap depth, the longitudinal and transverse energies must anticorrelate. In fact, this anticorrelation is apparent even in the no-laser simulation (Fig. 4g, Extended Data Fig. 3c). The simulations indicate that laser heating enhances the anticorrelation by displacing the population with the smallest total energies (Extended Data Fig. 3d). Qualitative, but non-trivial, agreements on these correlations between experiment (Fig. 4a–d) and simulation (Fig. 4e–h) further support our interpretation of three-dimensional cooling. Moreover, these correlation measurements provide opportunities for further studies of the cooling dynamics of dilute antihydrogen samples in a magnetic trap. Detailed understanding of the cooling dynamics will be important for future precision measurements, as the cooling process will define the initial condition of the anti-atom population in these measurements.

## 1S–2S spectroscopy with laser-cooled antihydrogen

**Fig. 5:**
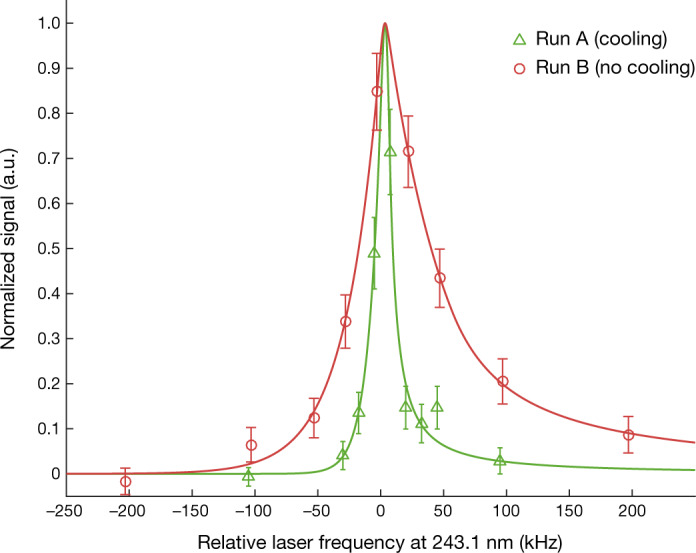
Recorded spectra of the 1S–2S transition from runs A and B. The fits use lineshapes informed by simulation (Methods). Both of the spectra are normalized to their fitted height and have a frequency-independent background subtracted to illustrate the difference in line shape. The subtracted background is 3.6 (18.3) annihilation events per bin in run A (B) and the fitted signal amplitude is 84.6 (135) events. The error bars represent 1 s.d. counting statistics. a.u., arbitrary units.

Finally, we studied the influence of laser cooling on measurements of 1S–2S transitions in the ‘spectroscopy’ experiment. In run A, we collected a sample of antihydrogen atoms for 9.1 h using the ‘stack and cool’ procedure, and then continued the 121.6-nm illumination for an additional 6 h (detuning at –220 MHz). This sample was then probed with 243.1-nm light with a set of nine discrete frequencies covering ±100 kHz around the expected 1S_d_–2S_d_ transition. The frequency was stepped after a 1-s exposure, alternately in ascending and descending order for a total of 100 s exposure at each frequency. For comparison, a sample of trapped antihydrogen not subjected to laser cooling was probed in a similar manner, this time with 2-s exposures spanning ±200 kHz around the 1S_d_–2S_d_ transition (run B). Figure 5 shows a comparison of the 1S_d_–2S_d_ transition spectral profiles between the laser-cooled (run A) and uncooled (run B) samples. In the plot, we have subtracted the frequency-independent background, which consists largely of annihilation events on background gas in the trap, as well as a small fraction of misidentified cosmic-ray events. The data were fit with a function that has been developed to match simulated 1S–2S spectra (Methods). The fitted linewidths are 57.6 ± 12 kHz (uncooled), and 14.4 ± 4.0 kHz (laser cooled), quantifying the degree of the line narrowing already clearly visible in Fig. 5.

It is worth noting that the 1S–2S spectral width is free of first-order Doppler broadening and is instead dominated by transit-time broadening. Thus, the width is sensitive to the velocity of antihydrogen perpendicular to the spectroscopy laser. As this motion is also nearly perpendicular to the cooling laser (Fig. 1a), the narrowing of the 1S–2S line width provides additional evidence that laser cooling on a single axis as implemented here results in three-dimensional cooling of the trapped atoms on the timescales studied here. The limited number of points in the current data sample does not allow for a more precise determination of the absolute frequency of the 1S–2S transition^12^. A more detailed analysis of the spectroscopy results will be presented in a future publication (C.J.B. et al., manuscript in preparation).

## Conclusions and future perspectives

In this Article, we have reported the demonstration of laser cooling of antihydrogen. The anti-atoms are cooled in three dimensions, with longitudinal cooling characterized by the narrowing of the 1S–2P lineshape and transverse cooling determined by the TOF method. In particular, the TOF diagnostics revealed strong growth of the transversely cold population; an order of magnitude reduction in the median transverse energy was observed—with a substantial fraction having submicroelectronvolt transverse kinetic energies. Furthermore, laser cooling was applied before probing the 1S–2S transition, resulting in a striking narrowing of its observed spectral width, and confirming transverse cooling. To achieve these results, the anti-atoms were held and exposed to Lyman-α and 243.1-nm radiation for up to 17 h in a single experiment, demonstrating ALPHA’s capability for robust and sustained laser cooling and spectroscopic operations.

Beyond this initial demonstration, we foresee a substantial reduction in the final energies of the anti-atoms and a notable increase in cooling rates. Our Lyman-α laser system is currently undergoing an upgrade to increase its pulse energy and repetition rate. The precise value of the detuning and the magnetic field configuration during the laser-cooling process have yet to be optimized, and schemes employing a time-dependent change of laser frequency and/or magnetic field have not yet been explored. It is noted, however, that in the limit of high cooling laser power, the indirect coupling between degrees of freedom will eventually limit the cooling rate in our current, one-dimensional laser access geometry. The use of laser cooling in conjunction with other cooling schemes, such as adiabatic expansion cooling^44^, should further reduce the antihydrogen energy. The diagnostic of pulsed laser probing and correlation studies reported in this Article makes these experimental optimizations viable. We also note that the ‘stack and cool’ procedure demonstrated here has a particular practical advantage; it transforms the antihydrogen accumulation period, currently hours of ‘dead time’ in the experimental cycle, into an efficient cooling period.

The implications of this work are both immediate and far reaching. Precision spectroscopic measurements on antihydrogen for tests of charge–parity–time invariance will be improved, as slower anti-atoms offer smaller Doppler, Zeeman and transit-time broadenings, and an increased excitation rate—all at the same time. For example, as reported in this Article, laser cooling has immediately resulted in a striking narrowing of the observed 1S–2S transition spectral width—a result that promises rapid progress towards matter-like precision^45^ in antihydrogen spectroscopy. It is now in principle possible to interrogate trapped antihydrogen atoms with lower velocities than those in the sample of hydrogen used in the current best measurements for matter^45^.

In addition, the determinations of the antihydrogen Lamb shift, fine-structure splitting^11^ and the hyperfine splitting^13^ will greatly benefit from the reduced Doppler, Zeeman or transit-time broadenings. Laser cooling will enhance the response of the anti-atoms to external fields; hence, it will have direct impact on the ongoing measurements of the gravitational acceleration^14^ and electric charge neutrality^46^ of antihydrogen. Very cold anti-atoms can be densely confined in a small volume, providing a nearly point-like source of antihydrogen. This could be used, for example, as a source for an anti-atomic fountain for interferometric measurements^47^. Furthermore, the creation of antimatter molecules^48,49^ may be possible in such an environment. Cooled anti-atoms could be confined with a weaker field from a non-superconducting or even a permanent magnet, simplifying many aspects of antihydrogen experiments. Finally, the techniques demonstrated here can be applied to simultaneous cooling of normal hydrogen and antihydrogen, which will enable direct comparison measurements—an ultimate goal of the field. Overall, laser-cooled antihydrogen will be a transformative tool in antimatter studies, with its most exciting applications probably yet to be dreamt of. At a minimum, it will be the starting point of our future precision measurements using magnetically trapped antihydrogen.

## Methods

### Related previous work on laser cooling

Magnetic trapping of atomic hydrogen has been pioneered by the groups at the Massachusetts Institute of Technology^27,50^ and Amsterdam^26,51^. Evaporative cooling^52^ was used to create hydrogen samples at ultracold temperatures in these experiments, but because of the required high densities, the technique does not apply to current antihydrogen experiments. In ref. ^26^, laser cooling of hydrogen to 11 mK was demonstrated over a timescale of 15 min in a high-density environment (about 10^11^ cm^−3^), by starting from 80-mK hydrogen precooled via evaporation. The cooling laser had one-dimensional access to the trapped atoms, and interatomic collisions were used to cool the other degrees of freedom. Pritchard’s group demonstrated laser cooling of magnetically trapped sodium atoms, also with one-dimensional laser access^36^. The observed three-dimensional cooling was attributed to the coupling of the degrees of freedom in the magnetic trap, as no density-dependent effects were observed in the experiment. Note, however, that the atomic densities (about 10^9^ cm^−3^) were some nine orders of magnitude larger than those in this work. See refs. ^53–55^ for other proposals for laser cooling of (anti)hydrogen. Antiproton trapping and electron cooling were first developed at CERN in the late 1980s^56,57^, and positron accumulation in a Surko-type buffer gas trap was developed by the University of California San Diego group^58^.

### Narrow-line pulsed Lyman-α laser system and lineshape measurements

Our Lyman-α laser system is based on the one used in our previous studies^10,11^ but with some improvements. The improvements include a new THG cell, a new 729.4-nm generation optical cavity with improved power and stability, and simultaneous monitoring of the pulse amplified 729.4-nm frequency via a Fabry–Pérot cavity, which allowed a measurement of frequency during the many hours of experiments.

Our Fabry–Pérot cavity had a free spectral range of 745 MHz, a finesse of 75 and a resolution of 10 MHz. To obtain the spectrum, we used a step-scan method, in which the Fabry–Pérot cavity was scanned with a frequency step of 3.5 MHz every one to two pulses.

The pulse energy was measured using a solar-blind photomultiplier detector, the sensitivity of which at 121.6 nm was calibrated by Hamamatsu Photonics. Throughout the laser irradiation on the trapped anti-atoms, waveforms of the photomultiplier output were sampled every two pulses, and were recorded into a digital oscilloscope, which in turn were analysed offline to determine the pulse energy. A typical shot-to-shot variation of the pulse energy is of the order of 20% (1 s.d.).

Our frequency uncertainty in the lineshape measurements (Fig. 2a) is estimated to be 54 MHz (1 s.d.) from a quadratic sum of the following effects: 729.4-nm cavity frequency drift (37 MHz), cavity frequency correction accuracy (18 MHz), wavemeter drift (30 MHz) and wavemeter offset (18 MHz). When multiple runs are added for the same series, the average of the frequencies is taken for each data point in Fig. 2a. See refs. ^37–39,59^ for other related work on Lyman-α lasers.

### Hyperfine-purified doubly polarized antihydrogen samples

The spectral linewidth of the 1S–2P transitions of antihydrogen in our (uncooled) conditions is of the order of 1 GHz. Hence the hyperfine splitting in the 1S–2P transitions (about 700 MHz) is not clearly resolved. This presents complications both in the cooling process and in its diagnosis in the cooling experiment (series 1–4). The expected optimal detuning frequency for laser cooling in our conditions is of the order of a few hundred megahertz. If we applied the laser at a frequency a few hundred megahertz red-detuned with respect to the 1S_c_ → 2P_a+_ transition to optimally cool the 1S_c_ state atoms, the 1S_d_ state atoms would ‘feel’ the blue-detuned light, and hence would be heated. In contrast, if we red-detuned the laser with respect to the 1S_d_ state atoms, it would be far red-tuned for the 1S_c_ atoms, and the overall efficiency of cooling would be reduced, making it more difficult to detect the effect of cooling with the small number of the anti-atoms we have. We address this issue by eliminating (with an efficiency of about 95%) the 1S_c_ atoms by application of resonant microwaves to drive the 1S_c_ → 1S_b_ transition^11,22^, which forces them out of the magnetic minimum trap. This results in a doubly spin-polarized, that is, hyperfine-purified, antihydrogen sample.

In the spectroscopy experiment (runs A and B) in which the 1S_d_–2S_d_ spectrum is probed, we did not perform this hyperfine purification. However, as the observed 1S–2S linewidth is orders of magnitude smaller than the hyperfine splitting, there is no risk of contamination with signal from less efficiently cooled atoms in the 1S_c_ state.

### SVD and machine learning analysis

Charged particles (mostly pions) from antihydrogen annihilations are detected by the SVD, consisting of three layers of double-sided microstrip sensors, with a total of 36,864 readout channels. By reconstructing the trajectories of the pions, the positions of the annihilations (vertices) were determined with a resolution of several millimetres^33^. Data acquisition in ALPHA is implemented using MIDAS (Maximum Integrated Data Acquisition System)^60^.

The time resolution of the annihilation detection is about 1 μs. The timing of the detected events recorded in the SVD readout system was cross-correlated with the time of the Lyman-α laser pulses using a continuously running time-stamping system. For long experimental runs that last several hours, it was necessary to correct for a drift of the internal clock in the SVD system. A timing measurement with a relative precision of the order of 10^−11^ (that is, microseconds in 10 h) was achieved by correlating the SVD system clock against an atomic clock in the timing system. The validity of the cross-correction was confirmed by verifying that the temporal patterns of random cosmic-ray events match each other in the two timing systems.

A machine-learning-based algorithm^34,35^ was developed to classify events from the SVD, to distinguish annihilation signals from the cosmic-ray background. As with previous ALPHA work, we used a boosted decision tree (BDT) for classification, with 14 event parameters^12^. Optimal selection criteria on the BDT discriminant threshold depend on the expected signal-to-noise ratio, which varies substantially across our four experimental phases. Therefore, separate criteria were chosen for the four phases. They are, in order of highest signal to background to lowest, the hyperfine polarization, release, probing and cooling/heating phases. These criteria were selected ‘blindly’, that is, without looking at the actual data, to mitigate potential bias.

The BDT classifier used was trained and validated using four separate datasets. These are sets of cosmic rays and a set of annihilation events, each of which are further divided into training and validation datasets. A total of 2.3 × 10^6^ cosmic event samples were collected by the detector with no annihilating antimatter particles in the trap. Signal samples with 3.3 × 10^5^ annihilation events were collected from the mixing of antiprotons and positrons; this is assumed to be completely pure. Extended Data Table 1 presents the efficiency for reconstructing annihilation events and the corresponding background (normalized to the number of recorded events) with our machine-learning-based analysis for the cooling experiment. Also shown is a condition on the axial position of the annihilation vertices, applied to select the events in each phase.

### The annihilation events and their characteristics

The counts of the annihilations detected by the SVD during the different phases in the cooling experiment are given in Extended Data Table 2. The annihilations in the probing phase are counted by requiring a condition that the event occurred within a time window of 0 to 3 ms after each Lyman-α laser pulse. The events in other phases were counted without this condition. The counts are corrected for the estimated efficiencies and the cosmic background. On the basis of consistency checks on these annihilation events, we estimate the uncertainty in the relative run-to-run normalization of the number of atoms to be of the order of ±20%. Some observations are in order.

The probability for 121.6-nm-laser-induced spin flips, deduced by the ratio of the numbers of detected atoms during the probing and the release phase *N*_probe_/(*N*_probe_ + *N*_release_), is roughly 85–95% (Extended Data Table 2), implying that most of the anti-atoms were detected in the probing phase, and only a relatively small fraction survived until the release phase. This suggests that the observed characteristics of the probed antihydrogen atoms (Figs. 2–4, Extended Data Fig. 2) are a good representation of the total sample.

The increased rate of the detected spin-flip transitions in the probing phase provides additional indirect evidence for cooling of the anti-atoms, as the colder atoms are expected to be excited more efficiently by the probe laser. If we crudely approximate the time evolution of the antihydrogen population as a single exponential, its rates—when normalized linearly by the probe laser pulse energy of 0.5 nJ—are larger for the cooling (about 4 × 10^−4^ s^–1^) and the ‘stack and cool’ series (about 7 × 10^−4^ s^–1^) than for the no-laser series (about 2 × 10^−4^ s^–1^). The spin-flip rates are expected to be mostly sensitive, in our experimental conditions, to the longitudinal velocity of the atoms, which affects the spectral strength due to the Doppler broadening.

Owing to the presence of the cosmic-ray background, it is difficult to detect small numbers of annihilations spread over the cooling phase, which lasts for several hours. However, using machine-learning techniques to analyse the SVD data, we do observe a signal above the background level, indicating loss of trapped antihydrogen. See *N*_cool_ in Extended Data Table 2 for the estimated number of atoms lost. These events were generally not in time coincidence with the laser pulses, and hence are not due to spurious optical pumping to untrapped magnetic sublevels. The possible causes for the continuous losses during the cooling phase include the annihilations with the residual gas (see below) and the escape of quasi-trapped atoms, that is, the anti-atoms whose kinetic energy exceed the trapping well but are temporarily trapped^20^. See below for the losses during the heating phase.

### Laser heating

Although evidence for Doppler ‘heating’ of antihydrogen was seen in the broadened spectral lineshape (Figs. 2a, 4d), additional evidence for heating is observed in the antihydrogen loss rate. The loss of antihydrogen during the heating phase was notably larger than the rest of the series. For example, the number of losses during the heating phase, normalized to the number of stacks, is about 3.2 ± 0.3, compared with the corresponding loss rate of about 1.3 ± 0.3 for the cooling series. (See *N*_cool_*/N*_stack_ in Extended Data Table 2) Here the errors are statistical. Also, the axial position of such annihilations during the heating phase (reconstructed by the SVD) are peaked at the edges of the magnetic trap. This is consistent with the expectation that heated atoms escape from the lowest points of the magnetic trapping walls. (These ‘holes’ exist near the trap edges at some azimuthal angles due to the destructive interaction between the radial field components of the octupole and the mirror coils^61^.) We note that the energy distribution of the heated sample depends critically on the details of the magnetic field profile near the top of the trapping well (from which the anti-atoms escape). The fact that our magnetic field model is probably not as accurate in those regions, compared with near the bottom of the trapping well, may partly explain some of the minor discrepancies (Figs. 2, 3) between the experiment and the simulation for the heating series.

### Cooling simulations

For all aspects of the cooling experiment, the simulations were performed as in refs. ^9–11^. The only new aspect of the simulation is the treatment of the atom recoil due to photon absorption and emission, which was implemented for the cooling/heating phase of the experiment as well as the probing phase. As with the simulation of Lyman-α absorption^10,11^, the probability for photon absorption is determined from the Doppler-shifted absorption profile, which includes the laser intensity at the position of the antihydrogen atom when a laser pulse occurs. At each time step, the occurrence of an absorption event is determined by generating a random number and comparing it with the calculated probability. If the absorption occurs, the antihydrogen velocity is changed by ${v}_{{\rm{r}}{\rm{e}}{\rm{c}}}\hat{{\bf{k}}}$, where $\hat{{\bf{k}}}$ is a unit vector along the direction of the propagation of the laser light, and *v*_rec_ is the recoil velocity from momentum conservation, *E*_photon_/(*m*_H_/*c*) = 3.26 m s^−1^. Here *E*_photon_ is the energy of the photon, *m*_H_ is the mass of antihydrogen and *c* is the speed of light. The expected direction of the photon emission in a transition from a |*m*| = 1 *P* state to an *S* state has a form 1 + cos2(*θ*), where *m* is the quantum number for the projection of the orbital angular momentum and *θ* is the angle relative to the magnetic field direction. The photon emission direction is simulated by randomly choosing cos(*θ*) using the rejection method with the function [1 + cos^2^(*θ*)]/2. The distribution is uniform in the azimuthal angle, and hence is chosen randomly between 0 and 2π. The antihydrogen velocity is then changed by $-{v}_{{\rm{r}}{\rm{e}}{\rm{c}}}\hat{{\bf{k}}}$. Note that the spontaneous photon emission generally heats the atoms in the transverse direction, and this heating has to be countered by transverse cooling induced by the anharmonic motional coupling in the trap.

In the simulation results presented in this work for the laser-cooling experiment, one input parameter, *W*_cool_, was adjusted to approximately match the experimentally observed TOF and lineshape distributions. Here *W*_cool_ is the total amount of energy injected inside the trap by cooling/heating laser (that is, the average laser pulse energy times the total number of pulses). Reasonably good qualitative agreements were obtained—throughout various comparisons in this work—by reducing *W*_cool_ by a factor of four compared with the experimental estimate. (For the series with more than one run, the experimental estimate was derived by the average of *W*_cool_ over the runs, weighed by their number of stacking, *N*_stack_.) The probe laser energy was not scaled. The specific origin of this difference is currently unknown, and for this work we take *W*_cool_ as an effective parameter that may incorporate effects such as incomplete modelling of the physical processes (for example, the initial conditions of the anti-atoms or collisions with the residual gases), mechanical imperfections in the experiment (for example, the position of coil windings) or inaccuracies in our estimate of the experimental parameters (for example, laser beam position or the radius). The discrepancy is unlikely due to a simple calibration issue in the pulse energy measurement, as scaling up the detection efficiency by a factor four would destroy the approximate agreement between the observation and the simulation for the rates of the probing transitions. (In fact, the comparison for the probing transition at present favours a slightly higher laser pulse energy than the measurement.) Recall that both cooling and probing transitions were driven by the same laser and detected by the same detector. Nonetheless, the need for the scaling in the simulation does not affect our main conclusions in this Article. Note that the effect of tuning this parameter on the kinetic energies of the cooled anti-atom can be highly nonlinear. For example, in the simulations for the ‘stack and cool’ series, the mean kinetic energy of the probed anti-atoms in the untuned simulation is only a factor 1.6 to 1.7 lower than that with the tuned simulation, despite the factor of four difference in *W*_cool_.

### Transverse energy reconstruction from TOF

A model that is largely independent of simulation details was developed for the cooling experiment to reconstruct the transverse kinetic energy of the antihydrogen atom, $(1/2){m}_{{\rm{H}}}({{v}_{x}}^{2}+{{v}_{y}}^{2})$, where *v*_*x*_ and *v*_*y*_ are the components of its velocity in the plane perpendicular to the trap axis (Fig. 1a). The transverse energy of the anti-atom, at the time of its spin-flip transition during the probing phase, is computed from the TOF, that is, the time delay between the anti-atom’s exposure to the radiation and its annihilation. During this period, which typically lasts a few milliseconds, a tiny fraction (a few nanoseconds) is spent on the excitation and spin-flipping de-excitation of its positron state. The vast majority of this time is taken up by the traversal from the laser path, where the spin-flipping takes place, to the inner vacuum wall, where the annihilation takes place. As the laser beam goes through the centre of the magnetic trap, is nearly parallel to the axis and has a radius much smaller than that of the inner vacuum wall (3.6 mm versus 22.2 mm), we consider all anti-atoms to be on axis at the start of their flight. During this flight, the spin-flipped, high-field-seeking anti-atom is accelerated outward by the magnetic minimum trap. A good approximation to the force experienced by the anti-atom is to consider only the idealized octupole and solenoidal components of the magnetic field, ignoring the mirror coil component. This is a justifiable simplification, as most anti-atoms are ejected around the region central in *z* (the longitudinal coordinate), far from the axial ends of the magnetic minimum trap where the mirror coils have substantial influence. The azimuthal dependence of the octupolar field is also negligible. These simplifications entail that the potential seen by the spin-flipped anti-atom is now purely a function of radial distance from the axis. With the assumption that the anti-atom starts its flight on axis (radius *r* = 0), ends its flight on the inner vacuum wall (*r* = 22.2 mm) and experiences a force that is only a function of *r*, we can reconstruct its starting transverse kinetic energy (that is, the anti-atom’s transverse energy at the time of spin flip) from its TOF by solving a simple one-dimensional equation of motion. The accuracy of this method can be tested by comparing in simulations the actual (‘true’) transverse energy with the reconstructed one. Extended Data Fig. 1 shows such a comparison for the ‘stack and cool’ series simulation, where a good overall agreement is observed. The reconstructed transverse energies agree with the true energies within 10% on average; on an event-by-event basis, we observe an r.m.s. deviation of about 30% for most of the reconstructed energy range, except at the lowest energies. Within these uncertainties, the TOF-based reconstruction method gives the transverse energy of the individual antihydrogen atom—a powerful tool to study antihydrogen dynamics in a magnetic trap. The reconstructed transverse energies are plotted in Fig. 3 and used to make event selection in Fig. 4. Their mean values in each run, denoted ${\bar{{\epsilon }}}_{{\rm{T}}}$, are shown and compared with the simulation in Extended Data Fig. 2 (see below).

### Longitudinal energy upper limit from lineshape

Reconstructing the longitudinal energies from the observed lineshape in the cooling experiment (Fig. 2a) involves greater uncertainties than the transverse energy reconstruction (above), owing to the limited number of the frequency points, and the existence of various non-Doppler broadening mechanisms. The latter include the natural width, the Zeeman broadening, the laser linewidth and the effect of the depletion in the sample comprising a small number of antihydrogen atoms with virtually no collisions. To gain qualitative insight without relying on the full simulation model, we derive an upper limit of the mean longitudinal energy ${\mathop{{\epsilon }}\limits^{ \sim }}_{{\rm{L}}}$ by making crude simplifying assumptions; we assume that the broadening is caused entirely by the Doppler effect and ignore other line-broadening mechanisms. If an atom is probed at a frequency offset Δ*f* from the resonance frequency, its longitudinal energy is then given by 1/2*m*_H_(Δ*f* × 𝜆)^2^, where 𝜆 = 121.6 nm is the Lyman-α light wavelength. Here we ignored the small 2.3° intersection angle between the laser beam and the trap axis. The upper limit ${\mathop{{\epsilon }}\limits^{ \sim }}_{{\rm{L}}}$, shown in Extended Data Fig. 2, is obtained by averaging this deduced energy over the lineshape by linearly interpolating between the measured frequency points. In simulation, the values of ${\mathop{{\epsilon }}\limits^{ \sim }}_{{\rm{L}}}$ evaluated from simulated lineshapes can be compared with the true mean energies ${\bar{E}}_{{\rm{L}}}$; the derived upper limits ${\mathop{{\epsilon }}\limits^{ \sim }}_{{\rm{L}}}$ are a factor of two to four higher than ${\bar{E}}_{{\rm{L}}}$, indicating the presence of other important line-broadening mechanisms. Nonetheless, the experimentally derived upper limits ${\mathop{{\epsilon }}\limits^{ \sim }}_{{\rm{L}}}$ qualitatively agree with those derived from simulations. Alternative analysis methods, for example, fitting a Gaussian to the lineshape and converting its width to the longitudinal energy assuming the Doppler effect, also lead to similar agreements between experiment and simulation, when the same methods are applied to both lineshapes.

### Evolution of the longitudinal and transverse energies across the different cooling/heating runs

In Extended Data Fig. 2, we illustrate the qualitative behaviour of the cooling/heating process across the different experimental series in the cooling experiment, by plotting the parameters, ${\tilde{{\epsilon }}}_{{\rm{L}}}$ and ${\bar{{\epsilon }}}_{{\rm{T}}}$, for the longitudinal and transverse motions (described above). The figure shows a good correlation between ${\mathop{{\epsilon }}\limits^{ \sim }}_{{\rm{L}}}$ and ${\bar{{\epsilon }}}_{{\rm{T}}}$, across the experimental runs, implying that both the longitudinal energy and transverse energy are reduced, as cooling is applied to the antihydrogen sample. Simulated data, analysed in the same way, follow the experimental trend, providing support to the validity of our analysis. Although it is based on simplifying assumptions, the analysis presented in Extended Data Fig. 2 provides additional evidence that cooling in both transverse and longitudinal degrees of freedom—that is, three-dimensional cooling—has been achieved. Note that this analysis applies to the ensemble averaged energies. See Fig. 4 for correlations at the subensemble level.

### Error estimate via the bootstrapping method for the cooling experiment

The statistical dispersion of the distribution of the antihydrogen annihilation events originating from laser-driven spin flips is not necessarily Poissonian, due to the nature of the dynamics of the trapped atoms in our magnetic trap. Therefore, the error associated with statistical properties (for example, the width of a lineshape spectrum), deduced from these annihilation distributions, are calculated via the bootstrapping method. Briefly, the set of annihilations, either experimental or simulated, is resampled by random drawing with replacement. The resampled dataset contains the same number of annihilations as the original set, but may contain multiple copies of the same annihilation, and may miss some annihilations entirely. The statistical property of interest (the spectral width in this example) is then calculated from the resampled set. By repeating the resampling process 1,000 times (producing 1,000 spectra), a 1,000-long list of the deduced values (the widths) is generated. This list is then sorted in ascending order, where the 159th and 841st entry are then taken as the 1*σ* (68% probability) range of the error estimate. This procedure was used in this Article to determine the errors for the width of the lineshapes, the mean of the TOF distributions, the fraction of the atoms below certain energies, and the mean and median of the reconstructed energies.

### Cooling versus loss of hot atoms

Here we consider the question of whether selective loss of hot atoms, rather than laser cooling, can feasibly explain our observations. In fact, we do expect some small loss of the hottest atoms from the trap due to the random nature of the atom recoil from photon emission. However, overall consistency of a variety of aspects of our experimental observations with our expectations and detailed simulations provide strong evidence against the loss of hot atoms being responsible for the observed reduction in energy. For example, we observe a factor of five to ten increase in the relative population of the coldest atoms in Fig. 3a. Although there are some uncertainties in cross-normalizing different runs (of the order of ±20%), it is clear that the low-energy peaks observed in Fig. 3a cannot be made to go away by considering solely the loss of hot atoms. Also, the detected annihilations during the cooling phase represent a small fraction of the total atoms (<25%; see *N*_cool_/(*N*_cool_ + *N*_probe_) in Extended Data Table 2), and hence the impact of the lost atoms—even under the assumption that they were somehow selected to be the hottest atoms—on the average energies of the surviving atoms would be limited. Recall that we observe a factor of two to four reduction in average energy and a factor of three to ten in median energy on cooling. Furthermore, as observed for the heating series, the loss of hot atoms tends to occur at the axial edges of the trap (that is, the ‘holes’ of the magnetic trap; see the ‘Laser heating’ section above). We do not see evidence for such localized annihilations in the cooling runs. Finally, comparing the rates of the depletion of antihydrogen samples when probed by the 121.6-nm laser, the observed depletion rates are faster for cooled samples, as discussed above. This implies that the density of cold atoms has increased. Note that evaporative cooling of our trapped antihydrogen is not possible due to its extremely low density.

### Radial versus azimuthal degree of freedom in the transverse plane

As discussed elsewhere, in the cooling experiment, the energies of the anti-atoms are analysed via TOF for the transverse motion, and via the spectral lineshape for the longitudinal motion. While our TOF analysis is essentially one-dimensional (in the radial direction), our comparison with simulation showed that the method provides a good representation of total (two-dimensional) transverse energy of the probed atoms (Extended Data Fig. 1). This implies that the azimuthal component of the transverse energy—hence, the angular momentum *L*—of the atom’s trajectory at the time of spin flip is small. This can be attributed to the fact that our probe laser beam is located near *r* = 0; as a result, an atom with large azimuthal energy cannot effectively intersect the probe beam due the centrifugal barrier. Note, however, that as *L* is not a strictly conserved quantity in our system on the timescale of many seconds^9,43^, an anti-atom that has a high *L* at one moment can end up with a small *L*, hence eventually encountering the beam.

Therefore, the TOF method provides a means for a good estimate of the total transverse energy for the probed atoms. Combined with the observed cooling in the longitudinal degree of freedom via the lineshape, our diagnostics provide evidence for three-dimensional cooling of antihydrogen atoms.

We cannot, however, experimentally exclude the possibility for a class of high azimuthal energy atoms whose trajectories never interact with the cooling or probing laser. Nonetheless, even if these atoms existed, given that only 5–15% of total anti-atoms survive until the release phase without being probed, they do not affect our conclusion for the majority of the atoms.

### Other possible systematic effects for the cooling experiment

Here we discuss other possible systematic effects in the cooling experiment, which may influence quantitative modelling of the cooling process. They do not, however, affect our main conclusion of the observation of laser-cooled antihydrogen.

#### Run-to-run variations

Although our sample size is limited, we observed a notable variation, beyond expected from statistical fluctuations, in the effect of cooling in the two runs with essentially the same experimental condition in the cooling series (see two yellow data points with error bars in Extended Data Fig. 2). Such a variation indicates the presence of some uncontrolled effects in our experiment. The cause of such variations will be investigated in future studies. However, it is reassuring that it will be possible to characterize the effect of cooling on a run-by-run basis, by probing a fraction of the antihydrogen sample by the spin-flip method described in this work. This will alleviate the possible concern about such fluctuations when cooling is used for future precision measurements.

#### Residual gas

During the experimental campaign, we observed evidence for some deterioration in the vacuum condition of our cryogenic trap. Under normal conditions, the lifetime of antihydrogen in our trap is estimated to be greater than 60 h on the basis of the observed loss rates^62^. The excellent vacuum is maintained primarily via cryo-pumping on cold electrode surfaces. In our experience, the effect of the cryo-pumping saturates after the trap is held cold for several weeks. This was the case for this work, which took place right before the shutdown of the ALPHA-2 experiment. During the course of our ten-day experimental campaign for the cooling experiment, the lifetime of the antiprotons in our Penning trap decreased from about 20,000 s to 10,000 s. For example, the ‘stack and cool’ run (series 4) performed on the final day of the cooling experiment lasted about 15 h, hence we may expect some losses due to collisions with the residual gas; the observed annihilation counts (Extended Data Table 2) suggest the loss of antihydrogen in this series may be larger by up to 30–40%, compared with earlier shorter runs. It should be noted, however, that the temperature of the residual gas should be at or higher than the electrode temperatures (5–8 K), that is, much greater than that of trapped antihydrogen atoms (a trap depth of less than about 0.5 K in temperature units). According to our theoretical estimates and simplified simulations of the loss process, collisions with the warm gas should not substantially change the energy distribution of the trapped anti-atoms; they more likely either knock the anti-atoms out of the trap or directly annihilate them. Hence, while the presence of the residual gas may have affected the number of the surviving anti-atoms, or the observed rate of cooling, it does not affect our main conclusion that the anti-atoms were cooled by the interaction with the laser. It is reassuring that the effect of cooling is observed both across the samples (Fig. 2) and within the samples (Fig. 4). We note that a provision exists in the ALPHA-2 apparatus, should it be necessary, to substantially improve the vacuum, by mechanically blocking the flow of gases from the room temperature part into the cryogenic part of the trap.

### Linewidth determination of 1S–2S spectra

The spectra are fitted with an asymmetric function, which has been found to give good fits to simulated lineshapes consisting of 63 frequency points and with two orders of magnitude more counts than the data. The simulations span a large range of laser power for both the 243.1-nm spectroscopy laser and the 121.6-nm cooling laser.

The fit function is constructed from a double exponential peak with independent widths on the blue and red sides. The blue tail transitions smoothly into a power function at a point determined by the fit. The whole function is then convoluted with a Gaussian, rounding off the otherwise infinitely sharp peak of the double exponential. The shape parameters to be fit to data are therefore: two exponential widths, the Gaussian width and the grafting point on the blue tail. Three additional parameters, which do not affect the width enter the fit to data: the amplitude and central frequency of the peak, as well as a constant background term, which has been suppressed in the figure.

The uncertainties on the extracted widths are a combination of two error sources: the statistical error, which is evaluated from the spread of fits to pseudo-data generated from the recorded counts and their statistical error, and a systematic error originating from the selection of probed frequencies. We estimate this ‘sampling error’ from the spread of many simulated spectra differing in the choice of probed frequencies. The relatively larger error on the width of the cooled spectrum (run A) results mainly from the paucity of data points in the resonance region.

Each of the error types were also evaluated with a bootstrap method, resulting in consistent values.

## Online content

Any methods, additional references, Nature Research reporting summaries, source data, extended data, supplementary information, acknowledgements, peer review information; details of author contributions and competing interests; and statements of data and code availability are available at 10.1038/s41586-021-03289-6.

## Figures and Tables

**Extended Data Table 1:**
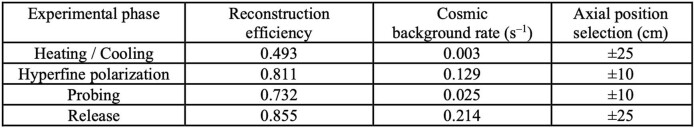
Annihilation detection efficiency and background for the cooling experiment

The efficiency for identifying an annihilation event via the machine learning algorithm for each phase of the laser-cooling experiment, and the corresponding background rate. The efficiency is normalized to the number of recorded detector events. Also shown is the selection criterion for the axial position of the annihilation events.

**Extended Data Table 2:**
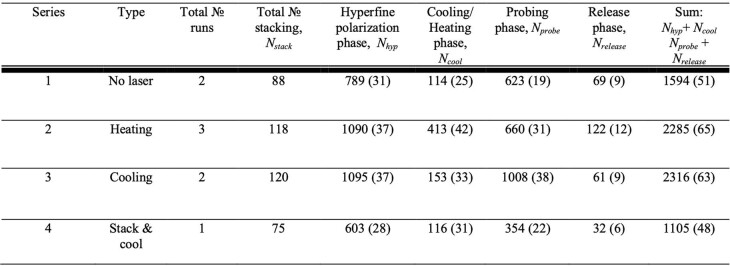
The number of antihydrogen atoms for the cooling experiment

Number of observed antihydrogen atoms—as defined by the number of detected annihilation events corrected for the efficiency and the background—are given for the different phases of the experiment and for each series. The numbers from the individual runs within the series are added. The events in the probing phase are time-gated with a window of 0 to 3 ms from the laser pulse. The 1 s.d. statistical counting errors are given in brackets. See Methods for a discussion.

## Data Availability

The datasets generated during and/or analysed during the current study are available from J.S.H. on reasonable request.
